# *catena*-Poly[[[di­aqua­bis­[1,2-bis­(pyridin-4-yl)diazene]copper(II)]-μ-1,2-bis­(pyridin-4-yl)diazene] bis­(perchlorate)]

**DOI:** 10.1107/S1600536813012269

**Published:** 2013-05-15

**Authors:** Ernesto Ballestero-Martínez, Cristian Saul Campos-Fernández, Victor Hugo Soto-Tellini, Simplicio Gonzalez-Montiel, Diego Martínez-Otero

**Affiliations:** a Escuela de Química, Centro de Electroquímica y Energía Química (CELEQ), Universidad de Costa Rica, 2060 San José, Costa Rica; b Centro de Investigaciones Químicas, Universidad Autónoma del Estado de Hidalgo, km. 4.5 Carretera Pachuca-Tulancingo, Col. Carboneras, Mineral de la Reforma, Hidalgo, CP 42184, Mexico

## Abstract

In the title compound, {[Cu(C_10_H_8_N_4_)_3_(H_2_O)_2_](ClO_4_)_2_}_*n*_, the coordination environment of the cationic Cu^II^ atom is distorted octa­hedral, formed by pairs of symmetry-equivalent 1,2-bis­(pyridin-4-yl)diazene ligands, bridging 1,2-bis­(pyridin-4-yl)diazene ligands and two non-equivalent water mol­ecules. The 1,2-bis­(pyridin-4-yl)diazene mol­ecules form polymeric chains parallel to [-101] *via* azo bonds which are situated about inversion centres. Since the Cu^II^ atom is situated on a twofold rotation axis, the monomeric unit has point symmetry 2. The perchlorate anions are disordered in a 0.536 (9):0.464 (9) ratio and are acceptors of water H atoms in medium–strong O—H⋯O hydrogen bonds with graph set *R*_4_^4^(12). The water mol­ecules, which are coordinated to the Cu^II^ atom and are hydrogen-bonded to the perchlorate anions, form columns parallel to [010]. A π–π inter­action [centroid–centroid distance = 3.913 (2) Å] occurs between pyridine rings, and weak C—H⋯O inter­actions also occur.

## Related literature

For the synthesis of *trans*-4,4′-azobispyridine, see: Brown & Granneman (1975 ▶). For the synthesis and structures of other polymers with Cu^I^ or Cu^II^ and *trans*-4,4′-azobispyridine, see: He *et al.* (2000 ▶); Kondo *et al.* (2006 ▶); Marinescu *et al.* (2010 ▶). For compounds with *trans*-4,4′-azobispyridine with other cations, including one with Zn^II^, see: Noro *et al.* (2005 ▶). For categorization of hydrogen bonds, see: Gilli & Gilli (2009 ▶). For a description of the Cambridge Crystallographic Database, see: Allen (2002 ▶). For the Hirshfeld test, see: Hirshfeld (1976 ▶); Spek (2009 ▶).
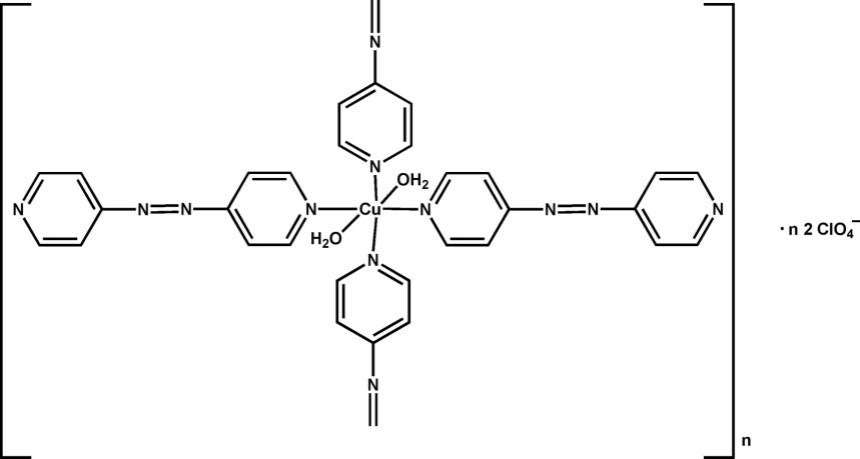


## Experimental

### 

#### Crystal data

[Cu(C_10_H_8_N_4_)_3_(H_2_O)_2_](ClO_4_)_2_
*M* *_r_* = 851.29
Monoclinic, 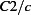

*a* = 20.5028 (6) Å
*b* = 9.5882 (4) Å
*c* = 18.7797 (6) Å
β = 96.629 (3)°
*V* = 3667.1 (2) Å^3^
*Z* = 4
Mo *K*α radiation
μ = 0.81 mm^−1^
*T* = 293 K
0.38 × 0.37 × 0.29 mm

#### Data collection

Oxford Diffraction Xcalibur Gemini diffractometer
Absorption correction: analytical [*CrysAlis PRO* (Oxford Diffraction, 2009 ▶) and Clark & Reid (1995 ▶)] *T*_min_ = 0.951, *T*_max_ = 0.965
27735 measured reflections
3738 independent reflections
2902 reflections with *I* > 3σ(*I*)
*R*_int_ = 0.026

#### Refinement

*R*[*F* ^2^ > 2σ(*F* ^2^)] = 0.043
*wR*(*F* ^2^) = 0.106
*S* = 2.67
3738 reflections
373 parameters
22 restraints
H atoms treated by a mixture of independent and constrained refinement
Δρ_max_ = 0.76 e Å^−3^
Δρ_min_ = −0.47 e Å^−3^

### 

Data collection: *CrysAlis PRO* (Oxford Diffraction, 2009 ▶); cell refinement: *CrysAlis PRO*; data reduction: *CrysAlis PRO*; program(s) used to solve structure: *SHELXS97* (Sheldrick, 2008 ▶); program(s) used to refine structure: *JANA2006* (Petříček *et al.*, 2006 ▶); molecular graphics: *OLEX2* (Dolomanov *et al.*, 2009 ▶) and *DIAMOND* (Brandenburg, 2010 ▶).; software used to prepare material for publication: *JANA2006* and *PLATON* (Spek, 2009 ▶).

## Supplementary Material

Crystal structure: contains datablock(s) global, I. DOI: 10.1107/S1600536813012269/fb2274sup1.cif

Supplementary material file. DOI: 10.1107/S1600536813012269/fb2274Isup2.mol

Structure factors: contains datablock(s) I. DOI: 10.1107/S1600536813012269/fb2274Isup3.hkl

Additional supplementary materials:  crystallographic information; 3D view; checkCIF report

## Figures and Tables

**Table 1:** Hydrogen-bond geometry (Å, °)

*D*—H⋯*A*	*D*—H	H⋯*A*	*D*⋯*A*	*D*—H⋯*A*
O2—H1*o*2⋯O1*b* ^i^	0.86 (3)	2.14 (3)	2.913 (12)	150 (3)
O2—H1*o*2⋯O3*a* ^i^	0.86 (3)	1.77 (3)	2.589 (10)	158 (3)
O1—H1*o*1⋯O2*a* ^ii^	0.86 (3)	2.21 (3)	2.969 (13)	147 (3)
O1—H1*o*1⋯O3*b* ^ii^	0.86 (3)	2.20 (3)	3.010 (11)	157 (3)
C5—H1C5⋯O1*b* ^iii^	0.93	2.51	3.346 (11)	149
C13—H1*c*13⋯O3*b* ^iv^	0.93	2.36	2.973 (11)	123

Symmetry codes: (i) 
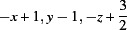
; (ii) 
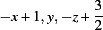
; (iii) 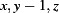; (iv) 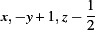.

**Fig. 1.**
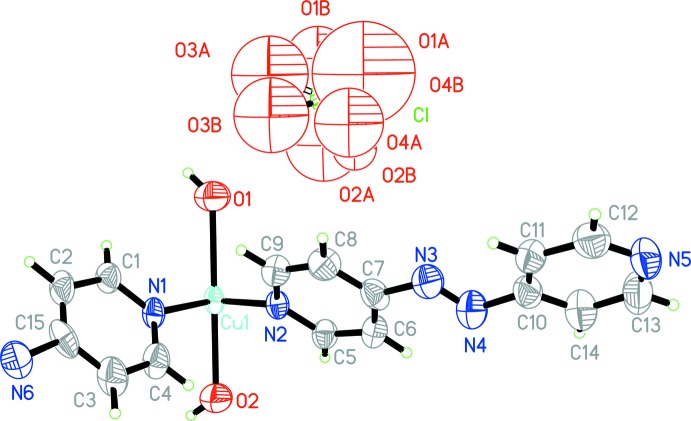
A view of the asymmetric unit of the title structure of catena-Poly[[[diaquabis[1,2-bis(pyridin-4-yl)diazene]copper(II)]-µ-1,2-bis(pyridin-4-yl)diazene] bis(perchlorate)] with the atomic labelling scheme. The displacement ellipsoids are shown at the 50% probability level.

**Fig. 2.**
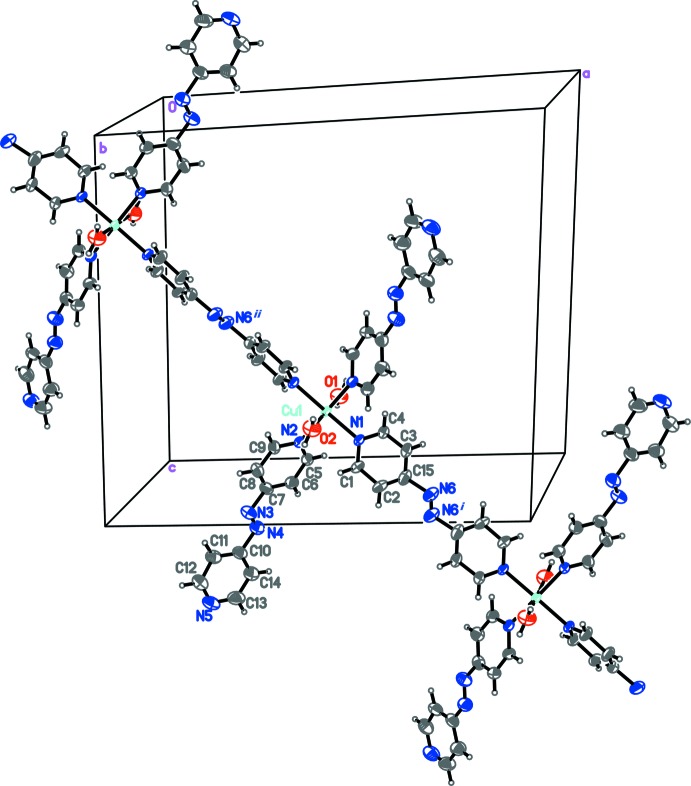
The cationic polymeric motif in crystal packing of the compound catena-Poly[[[diaquabis[1,2-bis(pyridin-4-yl)diazene]copper(II)]-µ-1,2-bis(pyridin-4-yl)diazene] bis(perchlorate)]. Symmetry codes (i) = -x + 1, y, z + 3/2; (ii) = -x + 3/2, -y + 1/2, -z + 2.

**Fig. 3.**
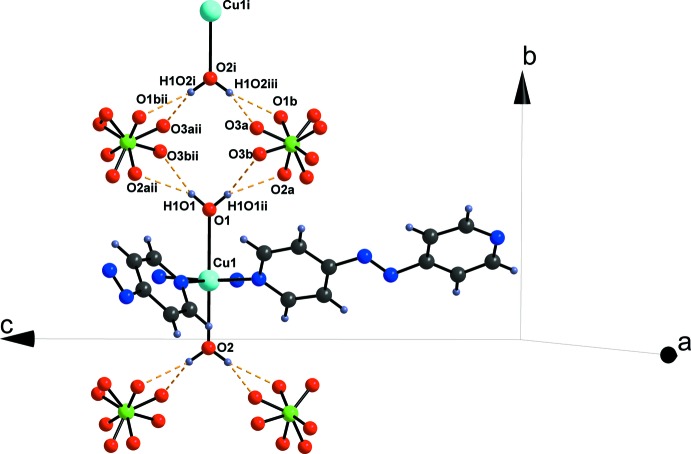
The intermolecular hydrogen bonds (O-H···O) donated by the water hydrogen atoms and accepted by the perchlorate O atoms. Symmetry codes: i: x, 1 + y, z; ii: 1 - x, y, 3/2 - z; iii: 1 - x, 1 - y, 3/2 - z. (See also Table 1).

**Fig. 4.**
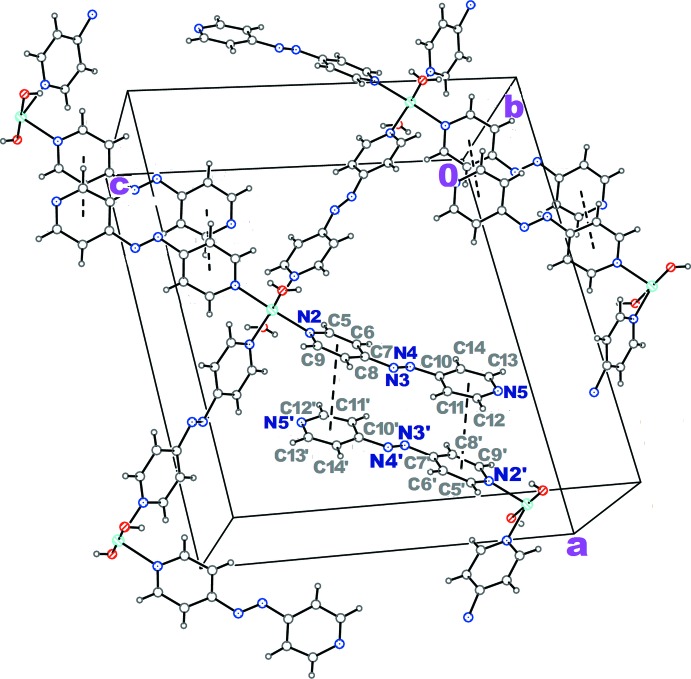
π-electron-ring—π-electron ring interaction between the rings N2//C5//C6//C7//C8//C9 (Cg1) and N5'//C13'//C14'//C10'//C11'//C12' (Cg2'). The primed atoms are related by the operation 3/2 - x, 1/2 - y, 1 - z.

## supplementary crystallographic information

### Comment

In our studies of new inclusion-coordination compounds with cyclodextrins, we have employed Cu(II) coordination compounds with 1,2-bis(pyridin-4-yl)diazene (Brown & Granneman, 1975). However, we obtained as a side product the title structure which is a new polymer formed by Cu(II) and 1,2-bis(pyridin-4-yl)diazene (Azpy), namely [Cu(C_10_N_4_H_8_)_3_(H_2_O)_2_]^2n+^]*_n_*^.^*2n*(ClO_4_)^-^ (Fig. 1). It is important to point out that the title structure is a product of a side reaction with no more than 5% yield. Water which was present in the reaction media allowed the crystallization of a polymeric structure with one-dimensional fishbone-type chain architecture (Fig. 2).

The polymeric motif present in the title structure is isomorphous to that of Zn-1D polymeric motif [Zn(C_10_N_4_H_8_)_3_(OH_2_)_2_]*_n_*^2n+^ which is present in *catena*-((µ_2_-1,2-bis(pyridin-4-yl)diazene-N,*N*')-diaqua-bis(1,2-bis(pyridin-4-yl)diazene-N)-zinc bis(hexafluorophosphate) bis(1,2-bis(pyridin-4-yl)diazene) tetrahydrate) (Noro *et al.*, 2005). However, this polymeric motif in the Zn-compound is partly disodered in the position which corresponds to the bond N6—N6^i^ (symmetry code i: -*x* + 3/2, -*y* + 1/2, 1 - *z*) in the title molecule (see below).

There are also known another related compounds that contain Cu(II), Azpy, water and other ligands in their metal coordination sphere such as tosylate (Kondo *et al.*, 2006) or molecules resulting from condensation reactions between 2,6-diformil-p-cresol with 1,3-diaminopropane and 2-aminoethyl-pyridine (Marinescu *et al.* 2010). Specifically [Cu(C_10_N_4_H_8_)_2_(H_2_O)_2_]^2n+^_n_ (He *et al.* 2000), which is present in *catena*-(bis(µ2-*trans*-4,4'-azopyridine-N,*N*')-copper dinitrate dihydrate, is a related 2-D motif, in which the Cu(II) is present in an octahedral enviroment: in its axial positions there are two water molecules, while in the equatorial plane is occupied by four Azpy ligands, this motif is also present in the title structure.

In the title compound, [Cu(C_10_N_4_H_8_)_3_(H_2_O)_2_]^2n+^]*_n_*^.^*2n*(ClO_4_)^-^, the cation Cu(II) is situated on the two fold-axis in the site 4*e*. The symmetry independent atoms are shown in Fig. 1. The coordination sphere of Cu(II) is octahedral and it is formed by pairs of the symmetry equivalent bis(1,2-bis(pyridin-4-yl)diazene, 1,2-bis(pyridin-4-yl)diazene and two non equivalent water molecules.

The 1,2-bis(pyridin-4-yl)diazene molecules form polymeric chains *via* azo-bonds formed between the symmetry equivalents of N6. These azo-bonds are situated about the inversion centres in 4*d* sites. These chains are parallel to (-1 0 1) (Fig. 2).

The perchlorate molecules are disordered and are acceptors of the water hydrogen bonds of a moderate strength. [For categorization of the hydrogen bonds, see Gilli & Gilli (2009).] The water molecules which are coordinated to Cu(II) form together with the perchlorates columns which are parallel to the monoclinic axis *b* (Fig. 3). The pertinent graph set motifs of the H atoms which are donated to the disordered perchlorate O atoms are *R*^4^_4_(12).

It is of interest that the bond length N3—N4 measures 1.209 (4) which is considerably longer than 1.166 (4) Å which is the distance between N6—N6^i^ (symmetry code i: -*x* + 3/2, -*y* + 1/2, -*z* + 2). This seems to be quite a large difference. On the other hand the adjacent bond-lengths C7—N3 and C10—N4 measure 1.448 (4) and 1.463 (4) Å while C15—N6 is 1.483 (3) Å long. The Cambridge Crystallographic Database (Allen, 2002; version CSD 5.33 with 586977 hits) has yielded 43 fragments of C_aryl_—N═*N*—C_aryl_ where N atoms are acyclic and are bonded just to two other atoms. The majority of the hits yielded N═N distances between 1.23–1.26 Å while the corresponding C—N distances were in the range 1.40–1.44 Å. There were other four hits with N═N distances between 1.17–1.19 Å while the corresponding C—C distances were between 1.47–1.51 Å. There is an indirect proportion between the C—N and N═N distances in the given fragment. Thus the present result is in accordance with the previous observations. However, it is worthwhile stressing that the pairs of atoms N3-C7, N6-C15 and C2-C15 are in disagreement with the Hirshfeld test (Hirshfeld, 1009) as indicate the alerts generated by *PLATON* (Spek, 2009).

Another interesting point are unequal Cu—O_water_ bond lengths: Cu—O1 and Cu—O1 are 2.403 (3) and 2.522 (3) Å despite of the fact that equatorial Cu—N1 and Cu—N2 are almost the same 2.035 (2) and 2.036 (2) Å. A search in the Cambridge Crystallographic Database (Allen, 2002; version CSD 5.33 with 586977 hits) has shown that such structures are found relatively rarely.

There is also present a π-electron ring···π-electron ring interaction in the structure (Fig. 4). It takes place between the rings N2//C5..C6//C7//C8//C9 and the symmetry equivalent of N5//C12//C11//C10//C14 related by the operation 3/2 - *x*, 1/2 - *y*, 1 - *z*. The distance between the pertinent centroids is 3.913 (2) Å (Spek, 2009). Moreover, there are also weak C—H···O interactions in the structure (see Table 1).

### Experimental

Trans-4,4'-azobispiridine was prepared according to literature method (Brown & Granneman, 1975). β-Cyclodextrin (1 mmol) was dissolved in 60 ml of water, *trans*-4,4'-azobispiridine (1 mmol) was added to this solution and the mixture was heated kept at 40 °C for 5 h approximately while stirring. After this time, the volume decreased to 44.0 ml. Then the solution was left to cool down to room temperature without stirring. An aliquot of 4.2 ml of 0.049 *M* aqueous solution of Cu(ClO_4_).6H_2_O (corresponding to 0.21 mmol of Cu(ClO_4_).6H_2_O) was slowly added without stirring to 22 ml of 0.0005 *M*β-cyclodextrin: *trans*-4,4'-azobispiridine water solution. Adding of Cu(ClO_4_).6H_2_O was stopped when precipitation of a violet powder appeared. Dark prismatic violet crystals suitable for X-ray analysis were obtained letting the solution to stand for three weeks. The longest dimensions of the obtained crystals varied between 0.2 - 0.5 mm. IR (KBr, cm-1): 3337 (*m*), 3099 (*m*), 1609 (*m*), 1589 (*m*), 1566 (w), 1413 (*m*), 1224 (w), 1098 (*s*), 845 (*m*), 622 (*m*), 571 (*m*), 624 (w).)

### Refinement

All the H atoms were discernible in the difference electron density map. The H atoms attached to the aryl carbons have been treated in the riding atom approximation with C—H =0.95 Å and *U*_iso_(H_aryl_)=1.2*U*_eq_(C_aryl_). Since both water molecules are situated on the crystallographic twofold axis only one symmetry independent water hydrogen is pertinent to each oxygen. Each of these H atoms have been restrained to the water O atoms by the distance restraint 0.860 (1) Å while *U*_iso_(H_water oxygen_)=1.5*U*_eq_(O_water oxygen_).

The O atoms of the perchlorate turned out to be disordered. The perchorate O atoms' electron density has been modelled by a split model in two positions with the constrained occupation of each of them. (The occupational parameters turned out to equal to 0.464 (9) and 0.536 (9).) The Cl—O distances were restrained to 1.440 (1) Å. The angles O—Cl—O were restrained to 109.47 (1)°.

### Crystal data

**Table:**

[Cu(C_10_H_8_N_4_)_3_(H_2_O)_2_](ClO_4_)_2_	*F*(000) = 1740
*M**_r_* = 851.29	*D*_x_ = 1.541 Mg m^−^^3^
Monoclinic, *C*2/*c*	Mo *K*α radiation, λ = 0.71073 Å
Hall symbol: -C 2yc	Cell parameters from 10880 reflections
*a* = 20.5028 (6) Å	θ = 3.1–26.3°
*b* = 9.5882 (4) Å	µ = 0.81 mm^−^^1^
*c* = 18.7797 (6) Å	*T* = 293 K
β = 96.629 (3)°	Prism, violet
*V* = 3667.1 (2) Å^3^	0.38 × 0.37 × 0.29 mm
*Z* = 4	

### Data collection

**Table:**

Oxford Diffraction Xcalibur Gemini diffractometer	3738 independent reflections
Radiation source: X-ray tube	2902 reflections with *I* > 3σ(*I*)
Graphite monochromator	*R*_int_ = 0.026
ω scans	θ_max_ = 26.4°, θ_min_ = 3.1°
Absorption correction: analytical [*CrysAlis PRO* (Oxford Diffraction, 2009) and Clark & Reid (1995)]	*h* = −25→25
*T*_min_ = 0.951, *T*_max_ = 0.965	*k* = −11→11
27735 measured reflections	*l* = −23→23

### Refinement

**Table:**

Refinement on *F*^2^	Primary atom site location: structure-invariant direct methods
*R*[*F*^2^ > 2σ(*F*^2^)] = 0.043	Secondary atom site location: difference Fourier map
*wR*(*F*^2^) = 0.106	Hydrogen site location: difference Fourier map
*S* = 2.67	H atoms treated by a mixture of independent and constrained refinement
3738 reflections	Weighting scheme based on measured s.u.'s *w* = 1/(σ^2^(*I*) + 0.0004*I*^2^)
373 parameters	(Δ/σ)_max_ = 0.044
22 restraints	Δρ_max_ = 0.76 e Å^−^^3^
57 constraints	Δρ_min_ = −0.47 e Å^−^^3^

### Fractional atomic coordinates and isotropic or equivalent isotropic displacement parameters (Å^2^)

**Table:**

	*x*	*y*	*z*	*U*_iso_*/*U*_eq_	Occ. (<1)
Cu1	0.5	0.24717 (4)	0.75	0.03398 (14)	
Cl	0.54953 (8)	0.75644 (13)	0.60764 (6)	0.0956 (5)	
N2	0.56205 (9)	0.25720 (18)	0.67295 (10)	0.0359 (6)	
N1	0.57826 (9)	0.24420 (19)	0.82723 (10)	0.0378 (6)	
C15	0.68446 (12)	0.2241 (3)	0.93132 (15)	0.0513 (9)	
C1	0.58515 (12)	0.3335 (3)	0.88204 (12)	0.0457 (8)	
H1c1	0.553612	0.402415	0.884613	0.0548*	
C2	0.63800 (13)	0.3267 (3)	0.93548 (13)	0.0531 (9)	
H1c2	0.641896	0.390084	0.973213	0.0637*	
N3	0.67222 (12)	0.3481 (3)	0.50343 (13)	0.0614 (9)	
C5	0.55314 (11)	0.1796 (3)	0.61320 (12)	0.0431 (8)	
H1c5	0.521918	0.108841	0.610531	0.0517*	
N6	0.74448 (13)	0.2009 (3)	0.98199 (13)	0.0652 (10)	
C6	0.58805 (13)	0.1996 (3)	0.55559 (14)	0.0503 (9)	
H1c6	0.580747	0.144085	0.514857	0.0604*	
C9	0.60886 (11)	0.3549 (3)	0.67708 (13)	0.0463 (9)	
H1c9	0.61685	0.406885	0.718993	0.0556*	
C4	0.62437 (11)	0.1462 (3)	0.82472 (13)	0.0525 (9)	
H1c4	0.620345	0.084669	0.7862	0.063*	
N4	0.66166 (12)	0.2783 (3)	0.44975 (14)	0.0626 (9)	
C11	0.74683 (13)	0.4261 (3)	0.39990 (14)	0.0571 (10)	
H1c11	0.762209	0.46114	0.444894	0.0685*	
C7	0.63456 (12)	0.3057 (3)	0.56043 (14)	0.0485 (9)	
C10	0.69675 (13)	0.3311 (3)	0.39178 (14)	0.0525 (10)	
C8	0.64559 (12)	0.3817 (3)	0.62202 (14)	0.0534 (10)	
H1c8	0.677626	0.450758	0.626648	0.064*	
C3	0.67725 (12)	0.1318 (3)	0.87591 (14)	0.0605 (11)	
H1c3	0.707548	0.060508	0.872858	0.0726*	
N5	0.75455 (13)	0.4230 (3)	0.27360 (13)	0.0724 (11)	
C12	0.77402 (15)	0.4687 (3)	0.33930 (17)	0.0666 (12)	
H1c12	0.80804	0.533335	0.345032	0.0799*	
C13	0.70650 (17)	0.3312 (4)	0.26799 (16)	0.0761 (13)	
H1c13	0.69199	0.29756	0.222456	0.0913*	
C14	0.67590 (16)	0.2808 (3)	0.32486 (16)	0.0681 (12)	
H1c14	0.642393	0.215145	0.317754	0.0817*	
O2	0.5	−0.0034 (3)	0.75	0.0688 (12)	
O1	0.5	0.5102 (3)	0.75	0.0725 (12)	
H1o2	0.4866 (17)	−0.055 (3)	0.7829 (14)	0.1033*	
H1o1	0.4862 (18)	0.567 (3)	0.7805 (16)	0.1088*	
O1a	0.5232 (6)	0.8609 (9)	0.5573 (5)	0.161 (7)	0.464 (9)
O2a	0.5038 (5)	0.6425 (9)	0.6076 (7)	0.238 (11)	0.464 (9)
O3a	0.5602 (4)	0.8164 (12)	0.6783 (3)	0.195 (5)	0.464 (9)
O4a	0.6109 (2)	0.7059 (14)	0.5874 (5)	0.188 (6)	0.464 (9)
O1b	0.4963 (4)	0.8525 (11)	0.6130 (6)	0.148 (4)	0.536 (9)
O2b	0.5246 (5)	0.6320 (6)	0.5712 (6)	0.115 (4)	0.536 (9)
O3b	0.5796 (6)	0.7209 (11)	0.6784 (3)	0.195 (6)	0.536 (9)
O4b	0.5976 (5)	0.8204 (13)	0.5679 (6)	0.314 (13)	0.536 (9)

### Atomic displacement parameters (Å^2^)

**Table:**

	*U*^11^	*U*^22^	*U*^33^	*U*^12^	*U*^13^	*U*^23^
Cu1	0.0240 (2)	0.0517 (3)	0.0246 (2)	0	−0.00415 (14)	0
Cl	0.1544 (12)	0.0710 (8)	0.0605 (6)	−0.0011 (9)	0.0092 (7)	0.0064 (6)
N2	0.0294 (9)	0.0468 (12)	0.0306 (10)	−0.0027 (8)	−0.0006 (8)	0.0010 (9)
N1	0.0280 (9)	0.0549 (12)	0.0288 (10)	0.0006 (9)	−0.0047 (8)	−0.0017 (9)
C15	0.0304 (13)	0.074 (2)	0.0460 (15)	−0.0075 (12)	−0.0122 (11)	0.0231 (14)
C1	0.0436 (14)	0.0534 (16)	0.0369 (13)	−0.0036 (12)	−0.0084 (11)	−0.0011 (12)
C2	0.0629 (17)	0.0571 (17)	0.0352 (14)	−0.0217 (14)	−0.0115 (12)	−0.0011 (12)
N3	0.0730 (16)	0.0581 (15)	0.0559 (15)	−0.0015 (12)	0.0198 (13)	0.0023 (12)
C5	0.0411 (13)	0.0502 (15)	0.0374 (14)	−0.0062 (11)	0.0025 (11)	−0.0021 (12)
N6	0.0789 (17)	0.0660 (18)	0.0487 (15)	−0.0019 (15)	−0.0011 (13)	0.0060 (11)
C6	0.0518 (15)	0.0614 (17)	0.0381 (14)	0.0020 (13)	0.0065 (12)	−0.0049 (13)
C9	0.0402 (13)	0.0532 (16)	0.0455 (15)	−0.0074 (12)	0.0049 (11)	−0.0033 (12)
C4	0.0367 (13)	0.079 (2)	0.0399 (14)	0.0137 (13)	−0.0047 (11)	−0.0051 (13)
N4	0.0620 (16)	0.0761 (18)	0.0512 (15)	−0.0010 (12)	0.0131 (12)	0.0004 (13)
C11	0.0693 (18)	0.0577 (17)	0.0465 (16)	0.0105 (15)	0.0160 (14)	−0.0007 (13)
C7	0.0470 (15)	0.0539 (15)	0.0472 (16)	0.0044 (13)	0.0167 (12)	0.0134 (13)
C10	0.0548 (16)	0.0570 (18)	0.0488 (16)	0.0103 (13)	0.0197 (13)	0.0080 (13)
C8	0.0500 (15)	0.0526 (17)	0.0596 (17)	−0.0114 (12)	0.0149 (13)	0.0005 (13)
C3	0.0384 (14)	0.090 (2)	0.0498 (16)	0.0141 (14)	−0.0076 (12)	0.0025 (16)
N5	0.0883 (19)	0.0797 (19)	0.0543 (16)	0.0033 (16)	0.0298 (14)	0.0107 (14)
C12	0.0715 (19)	0.0575 (19)	0.075 (2)	0.0012 (15)	0.0260 (17)	0.0043 (16)
C13	0.092 (2)	0.093 (3)	0.0451 (18)	0.001 (2)	0.0146 (17)	0.0001 (17)
C14	0.068 (2)	0.084 (2)	0.0537 (19)	−0.0058 (16)	0.0123 (15)	0.0019 (16)
O2	0.093 (2)	0.0504 (18)	0.065 (2)	0	0.0167 (17)	0
O1	0.089 (2)	0.0526 (19)	0.078 (2)	0	0.0163 (17)	0
O1a	0.281 (17)	0.078 (7)	0.097 (9)	−0.032 (9)	−0.083 (10)	0.037 (7)
O2a	0.35 (2)	0.111 (12)	0.283 (19)	0.030 (12)	0.177 (17)	0.059 (10)
O3a	0.328 (12)	0.178 (10)	0.077 (6)	0.159 (9)	0.012 (6)	−0.026 (6)
O4a	0.253 (8)	0.214 (14)	0.116 (7)	0.122 (9)	0.096 (7)	0.020 (6)
O1b	0.232 (9)	0.091 (5)	0.095 (7)	0.042 (6)	−0.086 (6)	−0.032 (5)
O2b	0.112 (5)	0.066 (5)	0.177 (10)	−0.016 (4)	0.051 (7)	−0.032 (6)
O3b	0.309 (15)	0.148 (9)	0.115 (7)	0.023 (9)	−0.029 (8)	−0.006 (6)
O4b	0.57 (3)	0.157 (13)	0.270 (19)	−0.102 (17)	0.26 (2)	−0.088 (12)

### Geometric parameters (Å, º)

**Table:**

Cu1—N2	2.0364 (19)	N4—C10	1.463 (4)
Cu1—N2^i^	2.0364 (19)	C11—H1c11	0.93
Cu1—N1	2.0350 (17)	C11—C10	1.368 (4)
Cu1—N1^i^	2.0350 (17)	C11—C12	1.385 (4)
Cu1—O2	2.403 (3)	C7—C8	1.363 (4)
Cu1—O1	2.522 (3)	C10—C14	1.368 (4)
N2—C5	1.341 (3)	C8—H1c8	0.93
N2—C9	1.337 (3)	C3—H1c3	0.93
N1—C1	1.334 (3)	N5—C12	1.327 (4)
N1—C4	1.337 (3)	N5—C13	1.316 (5)
C15—C2	1.378 (4)	C12—H1c12	0.93
C15—N6	1.483 (3)	C13—H1c13	0.93
C15—C3	1.361 (4)	C13—C14	1.386 (5)
C1—H1c1	0.93	C14—H1c14	0.93
C1—C2	1.391 (3)	O2—H1o2	0.86 (3)
C2—H1c2	0.93	O2—H1o2^i^	0.86 (3)
N3—N4	1.209 (4)	O1—H1o1	0.86 (3)
N3—C7	1.448 (4)	O1—H1o1^i^	0.86 (3)
C5—H1c5	0.93	Cl—O1a	1.439 (9)
C5—C6	1.378 (4)	Cl—O2a	1.440 (10)
N6—N6^ii^	1.166 (4)	Cl—O3a	1.439 (7)
C6—H1c6	0.93	Cl—O4a	1.440 (7)
C6—C7	1.390 (4)	Cl—O1b	1.440 (10)
C9—H1c9	0.93	Cl—O2b	1.440 (8)
C9—C8	1.372 (4)	Cl—O3b	1.440 (6)
C4—H1c4	0.93	Cl—O4b	1.440 (11)
C4—C3	1.370 (3)		
			
N2—Cu1—N2^i^	174.58 (7)	N3—N4—C10	111.9 (2)
N2—Cu1—N1	90.07 (7)	H1c11—C11—C10	120.8874
N2—Cu1—N1^i^	90.01 (7)	H1c11—C11—C12	120.8893
N2—Cu1—O2	92.71 (5)	C10—C11—C12	118.2 (3)
N2—Cu1—O1	87.29 (5)	N3—C7—C6	125.3 (2)
N2^i^—Cu1—N1	90.01 (7)	N3—C7—C8	115.3 (2)
N2^i^—Cu1—N1^i^	90.07 (7)	C6—C7—C8	119.4 (3)
N2^i^—Cu1—O2	92.71 (5)	N4—C10—C11	125.1 (2)
N2^i^—Cu1—O1	87.29 (5)	N4—C10—C14	115.7 (3)
N1—Cu1—N1^i^	178.40 (8)	C11—C10—C14	119.2 (3)
N1—Cu1—O2	89.20 (5)	C9—C8—C7	119.2 (2)
N1—Cu1—O1	90.80 (5)	C9—C8—H1c8	120.3901
N1^i^—Cu1—O2	89.20 (5)	C7—C8—H1c8	120.3912
N1^i^—Cu1—O1	90.80 (5)	C15—C3—C4	118.6 (3)
O2—Cu1—O1	180.0 (5)	C15—C3—H1c3	120.7243
C5—N2—C9	117.7 (2)	C4—C3—H1c3	120.7229
C1—N1—C4	117.74 (19)	C12—N5—C13	115.9 (3)
C2—C15—N6	126.8 (2)	C11—C12—N5	124.1 (3)
C2—C15—C3	119.5 (2)	C11—C12—H1c12	117.969
N6—C15—C3	113.7 (2)	N5—C12—H1c12	117.9667
N1—C1—H1c1	118.9506	N5—C13—H1c13	117.5416
N1—C1—C2	122.1 (2)	N5—C13—C14	124.9 (3)
H1c1—C1—C2	118.9493	H1c13—C13—C14	117.5406
C15—C2—C1	118.6 (2)	C10—C14—C13	117.6 (3)
C15—C2—H1c2	120.7066	C10—C14—H1c14	121.1841
C1—C2—H1c2	120.7056	C13—C14—H1c14	121.1852
N4—N3—C7	113.7 (2)	H1o2—O2—H1o2^i^	110 (3)
N2—C5—H1c5	118.4381	H1o1—O1—H1o1^i^	102 (3)
N2—C5—C6	123.1 (2)	O1a—Cl—O2a	109.5 (6)
H1c5—C5—C6	118.4384	O1a—Cl—O3a	109.5 (6)
C15—N6—N6^ii^	110.0 (2)	O1a—Cl—O4a	109.5 (7)
C5—C6—H1c6	121.132	O2a—Cl—O3a	109.5 (6)
C5—C6—C7	117.7 (2)	O2a—Cl—O4a	109.5 (6)
H1c6—C6—C7	121.1303	O3a—Cl—O4a	109.5 (5)
N2—C9—H1c9	118.6589	O1b—Cl—O2b	109.5 (6)
N2—C9—C8	122.7 (2)	O1b—Cl—O3b	109.5 (6)
H1c9—C9—C8	118.6588	O1b—Cl—O4b	109.5 (6)
N1—C4—H1c4	118.2663	O2b—Cl—O3b	109.5 (6)
N1—C4—C3	123.5 (2)	O2b—Cl—O4b	109.5 (7)
H1c4—C4—C3	118.2675	O3b—Cl—O4b	109.5 (6)

Symmetry codes: (i) −*x*+1, *y*, −*z*+3/2; (ii) −*x*+3/2, −*y*+1/2, −*z*+2.

### Hydrogen-bond geometry (Å, º)

**Table:**

*D*—H···*A*	*D*—H	H···*A*	*D*···*A*	*D*—H···*A*
O2—H1*o*2···O1*b*^iii^	0.86 (3)	2.14 (3)	2.913 (12)	150 (3)
O2—H1*o*2···O3*a*^iii^	0.86 (3)	1.77 (3)	2.589 (10)	158 (3)
O1—H1*o*1···O2*a*^i^	0.86 (3)	2.21 (3)	2.969 (13)	147 (3)
O1—H1*o*1···O3*b*^i^	0.86 (3)	2.20 (3)	3.010 (11)	157 (3)
C5—H1C5···O1*b*^iv^	0.93	2.51	3.346 (11)	149
C13—H1*c*13···O3*b*^v^	0.93	2.36	2.973 (11)	123

Symmetry codes: (i) −*x*+1, *y*, −*z*+3/2; (iii) −*x*+1, *y*−1, −*z*+3/2; (iv) *x*, *y*−1, *z*; (v) *x*, −*y*+1, *z*−1/2.
